# Activation of the eIF2α-ATF4 Pathway by Chronic Paracetamol Treatment Is Prevented by Dietary Supplementation with Cysteine

**DOI:** 10.3390/ijms23137196

**Published:** 2022-06-28

**Authors:** Valérie Carraro, Lydie Combaret, Cécile Coudy-Gandilhon, Laurent Parry, Julien Averous, Anne-Catherine Maurin, Céline Jousse, Guillaume Voyard, Pierre Fafournoux, Isabelle Papet, Alain Bruhat

**Affiliations:** 1 Université Clermont Auvergne, INRAE, UNH Unité de Nutrition Humaine, UMR1019, F-63000 Clermont-Ferrand, France; valerie.carraro@inrae.fr (V.C.); lydie.combaret@inrae.fr (L.C.); cecile.coudy-gandilhon@inrae.fr (C.C.-G.); laurent.parry@inrae.fr (L.P.); julien.averous@inrae.fr (J.A.); anne-catherine.maurin@inrae.fr (A.-C.M.); celine.jousse@inrae.fr (C.J.); pierre.fafournoux@inrae.fr (P.F.); 2 Université Clermont Auvergne, CNRS, Institut de Chimie de Clermont-Ferrand, F-63000 Clermont-Ferrand, France; guillaume.voyard@uca.fr

**Keywords:** acetaminophen, eIF2α-ATF4 pathway, GCN2, cysteine, liver, skeletal muscle, CARE-LUC mice

## Abstract

Chronic treatment with acetaminophen (APAP) induces cysteine (Cys) and glutathione (GSH) deficiency which leads to adverse metabolic effects including muscle atrophy. Mammalian cells respond to essential amino acid deprivation through the phosphorylation of the eukaryotic translation initiation factor 2α (eIF2α). Phosphorylated eIF2α leads to the recruitment of activating transcription factor 4 (ATF4) to specific CCAAT/enhancer-binding protein-ATF response element (CARE) located in the promoters of target genes. Our purpose was to study the activation of the eIF2α-ATF4 pathway in response to APAP-induced Cys deficiency, as well as the potential contribution of the eIF2α kinase GCN2 and the effect of dietary supplementation with Cys. Our results showed that chronic treatment with APAP activated both GCN2 and PERK eIF2α kinases and downstream target genes in the liver. Activation of the eIF2α-ATF4 pathway in skeletal muscle was accompanied by muscle atrophy even in the absence of GCN2. The dietary supplementation with cysteine reversed APAP-induced decreases in plasma-free Cys, liver GSH, muscle mass, and muscle GSH. Our new findings demonstrate that dietary Cys supplementation also reversed the APAP-induced activation of GCN2 and PERK and downstream ATF4-target genes in the liver.

## 1. Introduction

Acetaminophen (APAP) is the most commonly used drug for the management of pain and fever. It is the first-intention recommended medicine for the treatment of chronic pain, especially in older individuals, a growing population with a high risk of sarcopenia [1]. APAP is considered safe when used at standard doses. It is mainly metabolized in the liver into non-toxic glucuronide and sulfate conjugates. However, a small amount (4–10%) is converted by hepatic cytochrome P450 into the highly reactive compound N-acetyl-p-benzoquinone imine (NAPQI). NAPQI is detoxified by conjugation with the powerful intracellular antioxidant glutathione (GSH) leading to Cys and mercapturate conjugates [2,3]. Cysteine is the precursor of sulfate and GSH and the end-products of APAP detoxification processes are excreted along with the urine. Thus, APAP detoxification leads to a net loss of cysteine (Cys). Chronic treatment of adult rats with doses of APAP equivalent to the maximum posology for humans induces global Cys deficiency [4]. This is supported by a decrease in plasma concentration of free Cys, a decrease in GSH content in the liver, which is the detoxification site, but also in skeletal muscle which is atrophied [4]. Cys is a sulfur amino acid provided by dietary proteins and endogenously synthesized from methionine and serine. However, Cys becomes nutritionally essential when its endogenous synthesis is not sufficient to cover all metabolic needs [5]. Several cellular mechanisms could contribute to APAP-induced adverse metabolic effects that occur in the absence of hepatotoxicity. These mechanisms could be generated by the decrease in bioavailability of Cys or by oxidative stress consecutive to GSH decrease.

Mammalian cells have evolved complex signaling pathways that mediate the cellular response to a large number of stresses and a range of physiological changes and pathological situations. These pathways initiate a wide array of adaptative mechanisms and ultimately, if necessary, cell death. Reversible phosphorylation on serine 51 of the α subunit of eukaryotic translation initiation factor 2 (eIF2α) is a highly conserved regulatory event activated in response to diverse stresses [6]. In mammals, four eIF2α kinases act as early responders to disturbances in cellular homeostasis. One of these, GCN2 (general control nonderepressible 2) binds to deacylated transfer RNAs (tRNAs) to become active in response to amino acid deprivation [7,8]. Another one, PERK (PKR (RNA-dependent protein kinase)-like endoplasmic reticulum (ER) kinase) is activated by ER stress consecutive to accumulation of unfolded proteins in the ER, or perturbations in cellular energy, calcium homeostasis, or redox status [9,10]. The phosphorylation of eIF2α elicits a severe decline in de novo protein synthesis as its primary consequence. Accompanying this global translational control, it also favors increased translation of a few selected mRNAs containing short upstream open reading frames, including that of activating transcription factor 4 (ATF4) [11,12]. Once expressed, this transcription factor binds to C/EBP-ATF Response Element (CARE) sequences triggering increased transcription of a subset of specific target genes involved in metabolism, nutrient uptake, redox status, and regulation of apoptosis and autophagy [6,10,13,14]. ATF4 has been shown to be also required for the expression of genes that encodes mediators of muscle fiber atrophy most notably *Gadd45a* [15,16].

Although the eIF2α-ATF4 pathway has been shown to be activated in response to sulfur amino acid deficiency [17], the causal relationship between chronic treatment with APAP leads to cysteine deficiency and the activation of this signaling pathway has not been established. Therefore, our purpose was to study the activation of the eIF2α-ATF4 signaling pathway in response to APAP-induced Cys deficiency. To monitor the activation of this pathway in mice treated with APAP and identify the targeted organs, we used a bioluminescent transgenic model engineered in our laboratory. Then, the role of the eIF2α-kinase GCN2 and the effect of dietary supplementation with Cys on APAP-induced eIF2α-ATF4 pathway activation were studied in liver and muscle. Our main findings demonstrate that dietary Cys supplementation reversed the APAP-induced activation of GCN2 and PERK eIF2α kinases and the increase of ATF4-target genes mRNA.

## 2. Results

### 2.1. APAP Treatment Elicits the Induction of the eIF2α-ATF4 Pathway in Several Organs

**Figure 1:**
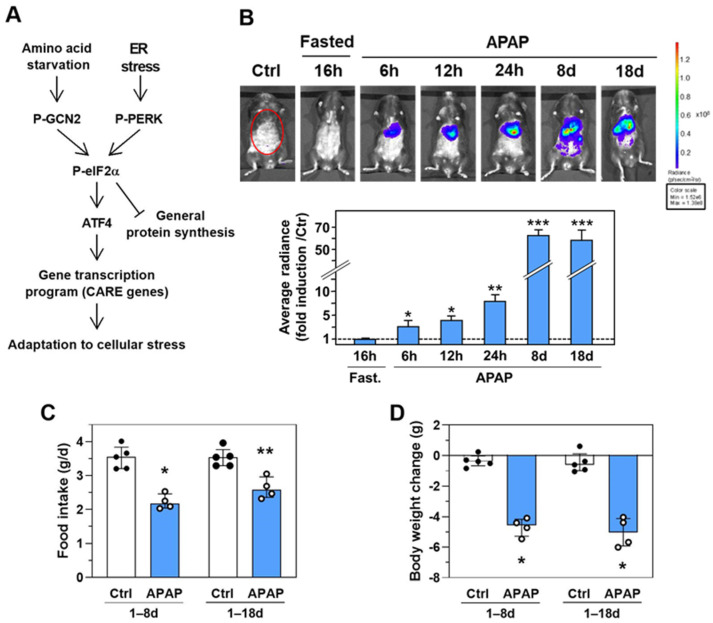
Visualization of the eIF2α-ATF4 pathway activation in response to APAP treatment. (**A**) Scheme of the eIF2α-ATF4 pathway. GCN2 is induced by essential amino acid starvation while PERK is activated by Endoplasmic Reticulum (ER) stress. These two kinases are active in their phosphorylated forms (P-GCN2 and P-PERK). The phosphorylation of eIF2α upregulates the translation of ATF4 mRNA. The transcription factor ATF4 enhances the transcription of many CARE-dependent genes that play a critical role in the adaptation to cellular stress. (**B**) CARE-LUC mice were fasted for 16 h and then fed a diet with 1% APAP (APAP) for 18 d *ad libitum*. Ventral views of representative mice are depicted at 6, 12, 24 h, 8, and 18 d. Photon emission was quantified using ROIs covering the abdominal area (red circles) as described in Materials and Methods. Results are given as the fold induction relative to the control (Ctrl) value. The graphs show means ± S.E.M. of 6 mice. * *p* ≤ 0.05; ** *p* ≤ 0.01; *** *p* ≤ 0.001 compared to the respective Ctrl value, Student’s *t*-tests. (**C**) Mean daily food intake for 1–8 d and 1–18 d of CARE-LUC mice receiving the Ctrl diet (Ctrl) or the APAP diet (APAP) *ad libitum*. (**D**) Body weight change during the experiment. In C and D graphs, each bar shows the median with interquartile, and dots are individual values. * *p* ≤ 0.05; ** *p* ≤ 0.01 vs. respective Ctrl; Mann Whitney test.

Activation of the eIF2α-ATF4 signaling pathway by GCN2 and PERK is recognized for integrating signals from different stresses leading to increased expression of specific CARE-regulated genes (Figure 1A). To visualize the activation of this signaling pathway in response to APAP treatment in the whole animal, we took advantage of a transgenic mouse line (CARE-LUC mouse) expressing the luciferase (LUC) reporter gene under the control of ATF4 which binds to CARE sequences [18]. These CARE-LUC mice enable the investigation of the eIF2α-ATF4 pathway activity over time in living animals and at the tissue level. Consumption of the diet with 1% APAP *ad libitum* resulted in a significant increase in bioluminescence intensity in the abdominal cavity from 6 h after ingestion. Bioluminescence intensity then increased sharply up to 8 d and remained very high at 18 d (Figure 1B). Mean daily food intake and body weight were significantly reduced over 8 and 18 d of APAP treatment (Figure 1C,D).

**Figure 2:**
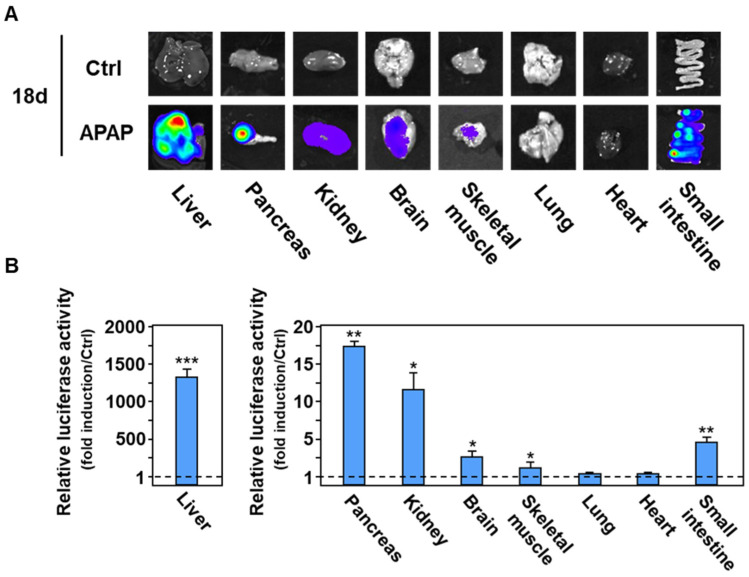
Identification of the eIF2α-ATF4 pathway target tissues in response to 18 d of APAP treatment. (**A**) Visualization of bioluminescence by imaging several organs. CARE-LUC mice were fed the Crtl diet (Ctrl) or the APAP diet (APAP) for 18 d. After bioluminescence imaging, mice were sacrificed by cervical dislocation, organs were rapidly excised and imaged, followed by snap-freezing of the organ for subsequent luciferase enzyme assay. (**B**) Luciferase activity measured in tissue extracts. Results are given as fold induction in APAP group relative to Ctrl group. The graphs show means ± S.E.M. of 6 mice. * *p* ≤ 0.05; ** *p* ≤ 0.01; *** *p* ≤ 0.001 compared to the respective Ctrl value, Student’s *t*-test.

To determine whether the eIF2α-ATF4 pathway becomes activated in the liver and muscle in response to 18 d-APAP treatment, we excised and individually imaged several organs from Ctrl and APAP-treated CARE-LUC mice. The liver was the most bioluminescent organ in response to APAP treatment and a middle induction of bioluminescence was visualized in skeletal muscle (Figure 2A). Induction of bioluminescence was also detected in the pancreas and some other studied tissues (brain, kidney, and small intestine). Quantification of the luciferase activity in organ homogenates confirmed the visualized bioluminescence (Figure 2B). Similar results were obtained at 8 d (Figure S1).

### 2.2. APAP-Induced Activation of the eIF2α-ATF4 Pathway in Liver and Skeletal Muscle Is Not Abrogated in the Absence of GCN2

**Figure 3:**
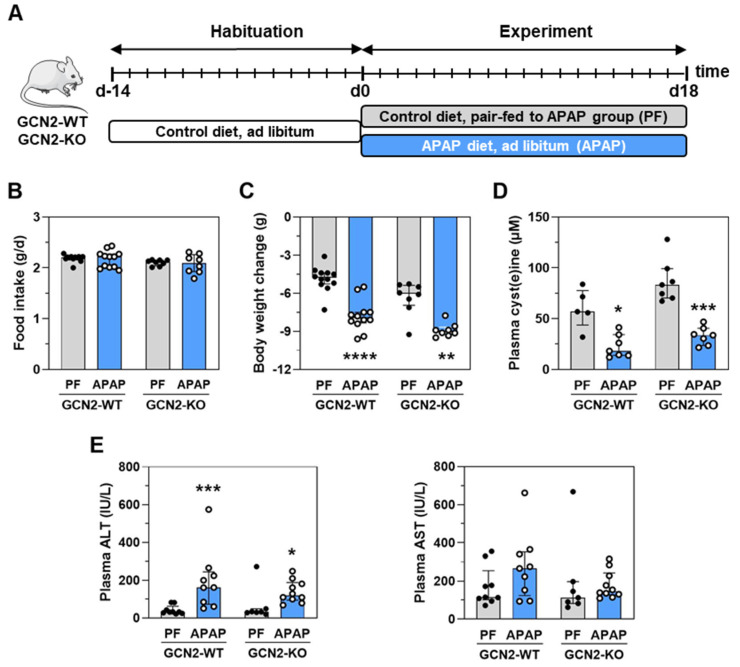
Effect of the absence of GCN2 in the response to treatment with APAP for 18 d. (**A**) *Gcn2* null (*Gcn2*^−/−^) or positive mice (*Gcn2*^+/+^) were fed the APAP diet (APAP) for 18 d or were pair-fed (PF) the Ctrl diet to match the daily food intake of the respective APAP-treated mice. (**B**) Mean daily food intake for 1–18 d. (**C**) Body weight change during the experiment. (**D**) Plasma concentration of cyst(e)ine (reduced form + homodimer + small heterodimers) at 18 d. (**E**) Plasma transaminase activities at 18 d. ALT: alanine aminotransferase AST: aspartate aminotransferase; IU: international unit. Each bar shows the median with interquartile and dots are individual values. * *p* ≤ 0.05; ** *p* ≤ 0.01; *** *p* ≤ 0.001; **** *p* ≤ 0.0001 vs. respective PF; Mann Whitney test.

The GCN2 kinase has been shown to be involved in the up-regulation of a gene expression program optimizing the cellular response to amino acid deprivation [19]. To explore GCN2’s role in the physiological adaptation to APAP-induced Cys deficiency in liver and skeletal muscle, we fed *Gcn2* null (*Gcn2*^−/−^) and positive (*Gcn2*^+/+^) mice the 1% APAP diet for 18 d (Figure 3A). Since APAP treatment significantly reduced food intake (Figure 1C), *Gcn2*^−/−^ and *Gcn2*^+/+^ control mice were presently pair-fed to respective APAP-treated mice (Figure 3B). APAP-induced body weight loss and plasma-free cyst(e)ine concentration decrease occurred in both genotypes (Figure 3C,D). APAP treatment slightly increased alanine aminotransferase (ALT) activity but had no effect on aspartate aminotransferase (AST) activity whatever the genotype (Figure 3E), meaning no major toxicity occurred.

**Figure 4:**
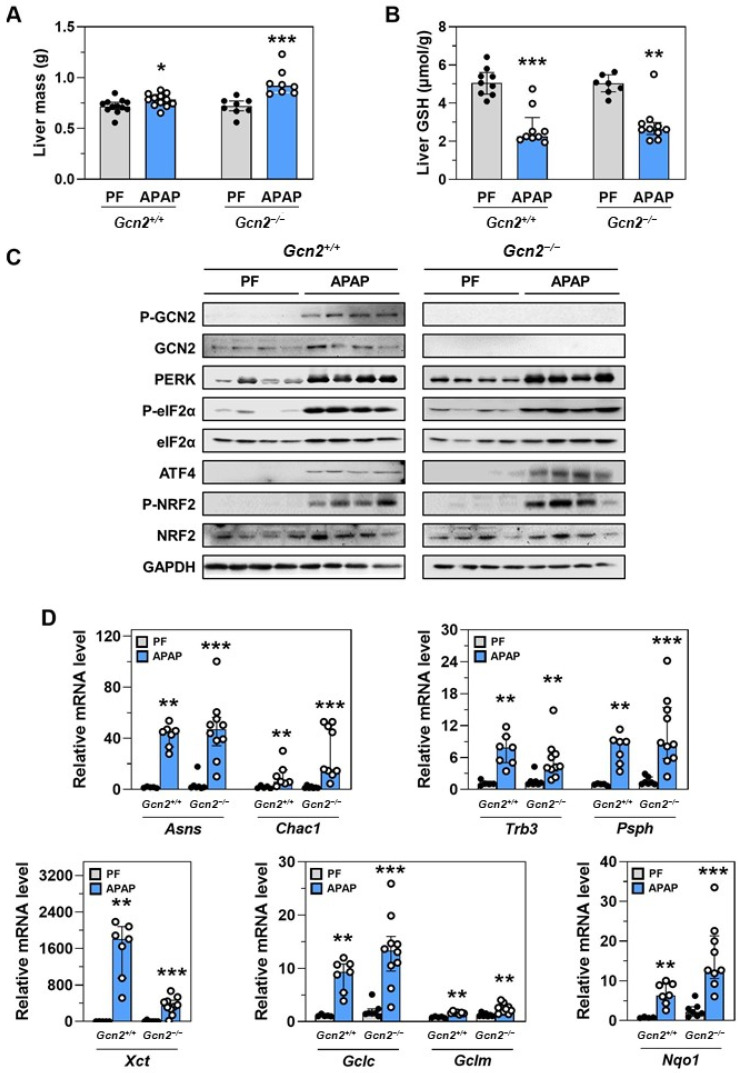
Impact of the absence of GCN2 in the APAP-induced up-regulation of the eIF2α-ATF4 pathway in the liver. *Gcn2* null (*Gcn2*^−/−^) or positive mice (*Gcn2*^+/+^) were fed the APAP diet (APAP) for 18 d or were pair-fed (PF) the control diet to match the daily food intake of the respective APAP-treated mice. (**A**) Liver mass. (**B**) Total free GSH (GSH, GSSG, and other small disulfides) concentration in the liver. (**C**) Immunoblot analysis of phosphorylated forms (P-) of GCN2, eIF2α, and NRF2, total GCN2, PERK and eIF2α, ATF4, NRF2, and GAPDH. (**D**) Expression of a set of ATF4-dependent and Nrf2-dependent genes. Total RNA extracted from the liver was analyzed by reverse transcription-quantitative PCR (RT-qPCR). In (**A**,**B**,**D**) graphs, each bar shows the median with interquartile, and dots are individual values. * *p* ≤ 0.05; ** *p* ≤ 0.01; *** *p* ≤ 0.001 vs. respective PF; Mann Whitney test.

The key role of the liver in APAP detoxification and the highest bioluminescence signal measured in response to the chronic treatment with APAP (Figure 2B) prompted us to study the role of GCN2 kinase in the up-regulation of the eIF2α-ATF4 pathway in this organ, firstly. Consumption of the APAP diet for 18 d resulted in higher liver mass and lower liver concentration of GSH in both genotypes (Figure 4A,B). Analysis of protein expression in the liver of *Gcn2*^+/+^ mice revealed that chronic treatment with APAP activated not only GCN2 but also PERK eIF2α kinase (visualized by the shift of the PERK band) with a subsequent increase in eIF2α phosphorylation and ATF4 protein abundance (Figure 4C). ER stress-induced PERK being presently activated in the absence of GCN2, levels of P-eIF2α and ATF4 remained increased in the liver of APAP-treated *Gcn2*^−/−^ mice.

Activation of UPR (Unfolded Protein Response) marker genes, notably Xbp1 mRNA splicing, confirmed the induction of ER stress in response to APAP in the liver (Figure S2). In addition, the phosphorylation of NRF2, an ATF4 binding partner involved in the oxidative stress response, was also increased in both genotypes (Figure 4C). The expression of ATF4-dependent genes such as *Asns*, *Chac1*, and *Trb3* confirmed the functional activation of the eIF2α-ATF4 pathway in the liver (Figure 4D). Of note, other ATF4/NRF2-dependent genes involved in serine synthesis (Psph), GSH metabolism (Gclc, Gclm), or cystine transport (Xct) were also induced by the chronic treatment with APAP (Figure 4D). Similar results were observed for the expression of the NRF2 canonical target gene Nqo1. In spite of the absence of GCN2, APAP treatment increased mRNA levels of all these genes (Figure 4D), as well as PERK, eIF2α, and NRF2 phosphorylation and ATF4 protein levels (Figure 4C). Thus, both GCN2 and PERK kinases are activated by the chronic treatment with APAP in the liver, GCN2 being not essential for the APAP-induced activation of the eIF2α-ATF4 pathway.

**Figure 5:**
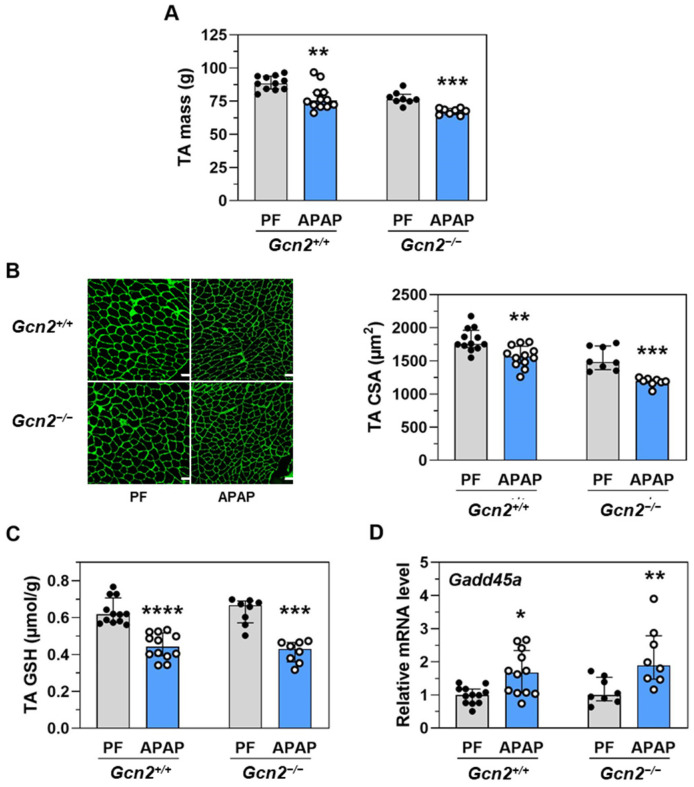
Impact of the absence of GCN2 on the response of the tibialis anterior (TA) muscle to APAP treatment for 18 d. *Gcn2* null (*Gcn2*^−/−^) or positive mice (*Gcn2*^+/+^) were fed a diet with 1% APAP (APAP) for 18 d or were pair-fed (PF) the respective control diet to match the level of food intake of the APAP-treated mice. (**A**) Mass of the two TA muscles. (**B**) Cross-sectional area (CSA) of muscle fibers. Scale bar = 50 µm. (**C**) Total free GSH (GSH, GSSG, and other small disulphides) concentration in TA muscle. (**D**) Expression of the ATF4-dependent gene *Gadd45*. Total RNA extracted from the liver was analyzed by reverse transcription RT-qPCR. Each bar shows the median with interquartile and dots are individual values. * *p* ≤ 0.05; ** *p* ≤ 0.01; *** *p* ≤ 0.001; **** *p* ≤ 0.001 vs. respective PF; Mann Whitney test.

Since the induction of bioluminescence was also detected in skeletal muscle in response to APAP treatment (Figure 2), we studied the role of GCN2 in the tibialis anterior (TA) skeletal muscle. TA mass and cross-sectional area (CSA) were lower in APAP-treated groups compared to pair-fed groups, in both *Gcn2*^+/+^ and *Gcn2*^−/−^ mice (Figure 5A,B). Whatever the genotype, the total GSH concentration was decreased in TA by the chronic treatment with APAP (Figure 5C) while the mRNA level of *Gadd45a*, an ATF4-dependent gene that encodes a mediator of muscle fiber atrophy, was increased (Figure 5D). As observed in the liver, GCN2 is not essential in muscle wasting induced by the chronic treatment with APAP.

### 2.3. APAP-Induced Activation of the eIF2α-ATF4 Pathway Is Reversed by a Dietary Supplementation with Cys

**Figure 6:**
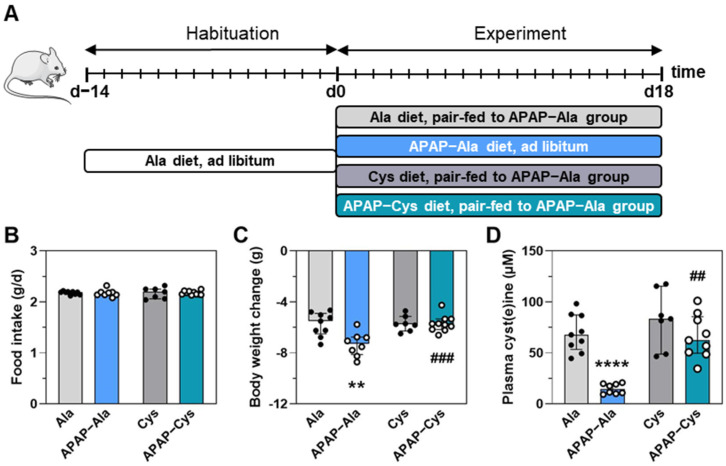
Effect of the dietary supplementation with Cys on the response to treatment with APAP for 18 d. (**A**) Experimental design: Ctrl and APAP diets were supplemented with alanine (Ala) or cysteine (Cys). Ala was added to make diets iso-nitrogenous to Cys diets. The APAP-Cys group was fed *ad libitum*. The three other groups were pair-fed with the APAP-Cys group. (**B**) Mean daily food intake for 1–18 d. (**C**) Body weight change during the experiment. (**D**) Plasma cyst(e)ine concentration at 18 d. Each bar shows the median with interquartile and dots are individual values. ** *p* ≤ 0.01; **** *p* ≤ 0.0001 vs. Ala; ## *p* ≤ 0.01; ### *p* ≤ 0.001 vs. respective Ala group; Mann Whitney test.

Dietary supplementation with Cys has been shown to reduce the deleterious effects of chronic treatment with APAP [20]. To explore the role of Cys deficiency in upregulating the eIF2α-ATF4 pathway, Ctrl and APAP diets were both supplemented with 0.5% Cys or 0.37% alanine so that the four diets were iso-nitrogenous (Figure 6A). Mean daily intake was similar in the four groups due to pair-feeding (Figure 6B). APAP-induced body weight loss and plasma cyst(e)ine concentration decrease were abrogated by the dietary supplementation with Cys (Figure 6C,D).

**Figure 7:**
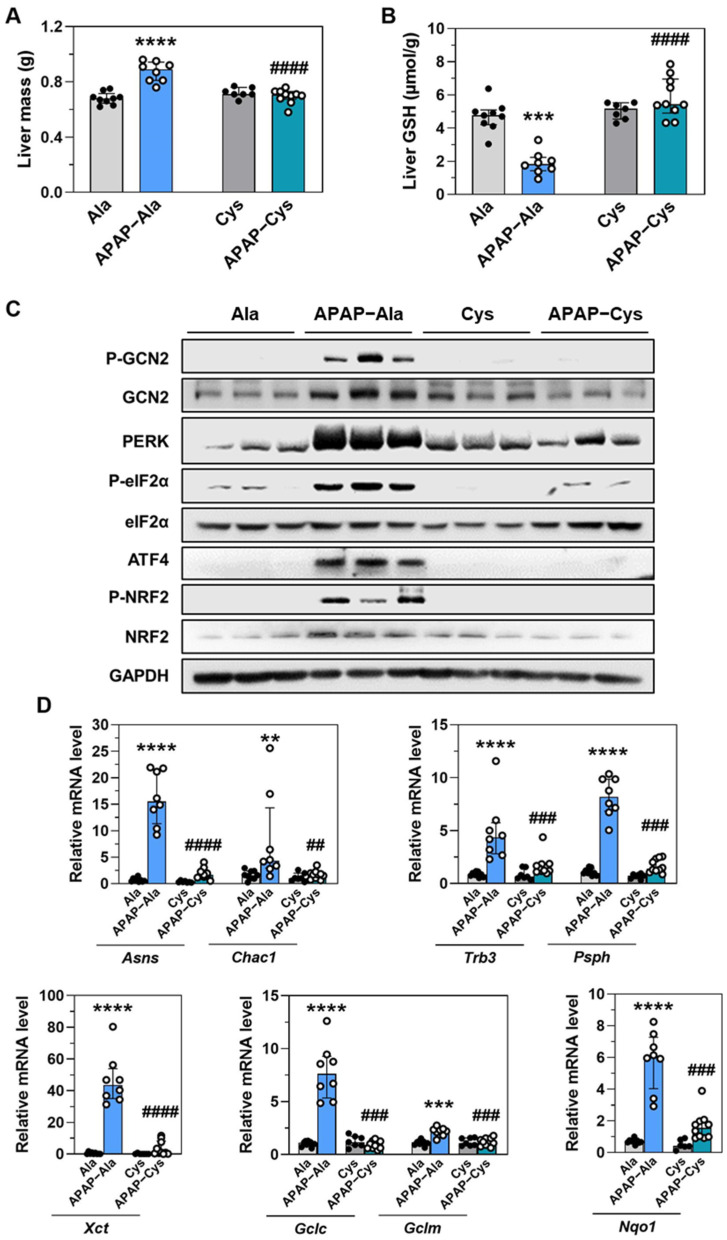
Effect of dietary supplementation with Cys on the liver response to treatment with APAP for 18 d. Ctrl and APAP diets were supplemented with alanine (Ala) or cysteine (Cys). Ala was added to make diets iso-nitrogenous to Cys diets. The APAP-Cys group was fed *ad libitum*. The three other groups were pair-fed with the APAP-Cys group. (**A**) Liver mass. (**B**) Total GSH concentration in the liver. (**C**) Immunoblot analysis of phosphorylation (P-) of GCN2, eIF2α, and NRF2, GCN2, PERK, eIF2α, ATF4, NRF2, and GAPDH. (**D**) Expression of a set of ATF4-dependent genes. Total RNA extracted from the liver was analyzed by reverse transcription RT-qPCR. Each bar shows the median with interquartile and dots are individual values. ** *p* ≤ 0.01; *** *p* ≤ 0.001 **** *p* ≤ 0.0001 vs. PF-Ala; ## *p* ≤ 0.01; ### *p* ≤ 0.001; #### *p* ≤ 0.0001 vs. respective Ala group; Mann Whitney test.

In the same line, the dietary supplementation with Cys prevented APAP-induced liver mass increase and GSH concentration decrease (Figure 7A,B). Analysis of protein expression in the liver revealed that supplementation with Cys abolished the activation of both GCN2 and PERK and the increase in eIF2α and NRF2 phosphorylation and ATF4 protein abundance (Figure 7C). The APAP-induced up-regulation of ATF4-dependent studied genes was reversed by the dietary supplementation with Cys (Figure 7D).

**Figure 8:**
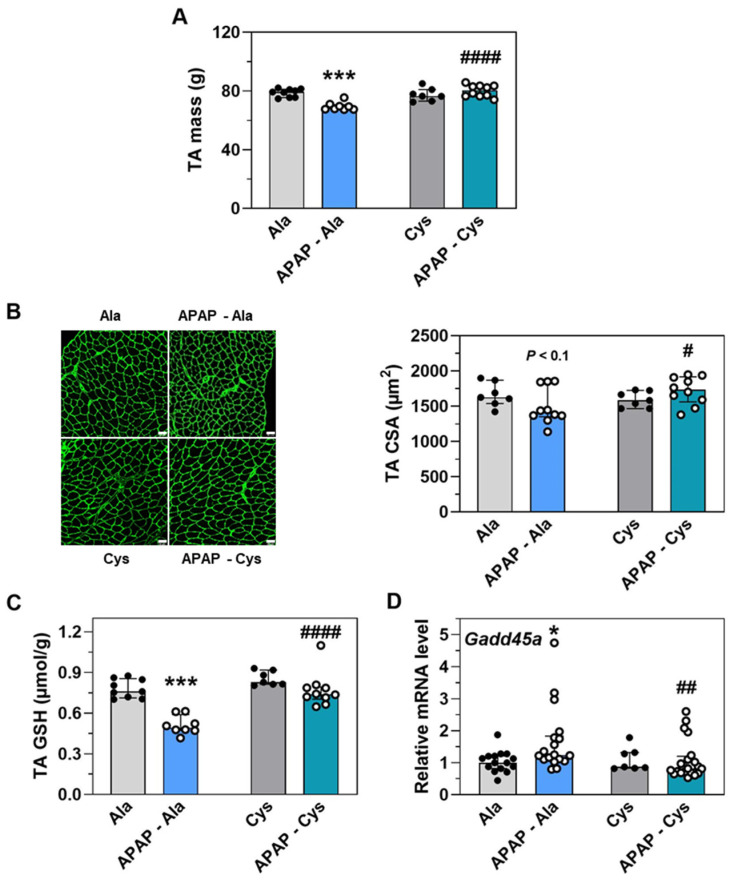
Effect of dietary supplementation with Cys on the response of tibialis anterior (TA) muscle to treatment with APAP for 18 d. Ctrl and APAP diets were supplemented with alanine (Ala) or cysteine (Cys). Ala was added to make diets iso-nitrogenous to Cys diets. The APAP-Cys group was fed *ad libitum*. The three other groups were pair-fed with the APAP-Cys group. (**A**) Mass of the two TA muscles. (**B**) Cross-sectional area (CSA) of muscle fibers. Scale bar = 50 µm. (**C**) Total GSH concentration in the TA. (**D**) Expression of the ATF4-dependent gene *Gadd45*. Total RNA extracted from the liver was analyzed by reverse transcription RT-qPCR. Each bar shows the median with interquartile and dots are individual values. * *p* ≤ 0.05; *** *p* ≤ 0.001 vs. PF-Ala; # *p* ≤ 0.05; ## *p* ≤ 0.01; #### *p* ≤ 0.0001 vs. respective Ala group; Mann Whitney test.

At the skeletal muscle level, the dietary supplementation with Cys prevented TA mass and CSA decreases (Figure 8A,B), and abrogated APAP-induced GSH concentration decrease, and Gadd45a mRNA increase (Figure 8C,D).

Taken together, these data demonstrated that dietary Cys supplementation abrogated APAP-induced activation of the eIF2α-ATF4 pathway as well as APAP-induced adverse metabolic effects in liver and skeletal muscle.

## 3. Discussion

APAP is a widely used analgesic and antipyretic drug, safe when used at standard doses and for the shortest period possible. However, overdose leads to hepatotoxicity and even acute liver failure [21], and long-term treatments at the upper standard therapeutic doses had been shown to be associated with adverse events [22]. Our previous studies shed light on muscle wasting in response to chronic treatment with APAP inducing Cys/GSH deficiency without being toxic [23]. Here we further show for the first time that the eIF2α-ATF4 signaling pathway is up-regulated in response to chronic treatment with a non-toxic dose of APAP, which induced Cys and GSH deficiency. GCN2 and PERK eIF2α kinases were both activated in the liver along with an increased transcription of adaptive genes downstream of eIF2α-ATF4 signaling. The absence of GCN2 did not impact the APAP-induced transcription of these genes in the liver or skeletal muscle. The dietary supplementation with Cys prevented not only the activation of the eIF2α-ATF4 signaling pathway in liver and skeletal muscle but also APAP-induced adverse metabolic events.

APAP-related signaling has mainly been investigated in the context of acute hepatotoxicity. Drug-induced liver injury usually manifests as elevations in transaminases three or more times above the upper limit of normal [22]. In the present study, AST activity was not significantly increased and the increase in ALT activity was far below the outsized increase when APAP induces acute liver toxicity (Figure 3) [24,25]. The present lack of toxicity is also supported by the fact that the mRNA encoding the pro-apoptotic transcription factor C/EBP homologous protein (CHOP) was not increased in the liver of mice chronically treated with APAP (Figure S3). CHOP has been shown to play a pro-damage role in response to APAP intoxication. Indeed, APAP strongly induced the *Chop* expression, and *Chop* knocked-out mice were protected from APAP-induced mortality and liver damage [26]. Although Chop mRNA is not increased, we presently report a progressive activation of the eIF2α-ATF4 signaling pathway during the first 24 h of consumption of a 1% APAP diet, corresponding to the maximum dose for a human, and higher activation during the chronic treatment over 8–18 d in mice abdomen. This activation occurred mainly in the liver, the site of APAP detoxification.

Previous studies and our data show that chronic treatment with APAP decreased liver GSH [4]. The APAP-induced decrease in hepatic GSH is basically interpreted as the irreversible loss of GSH after conjugation with NAPQI and its elimination as mercapturates in the urine [21]. In acute toxicity, GSH is also used for peroxynitrite scavenging [27]. Our data reveal, for the first time in vivo, other potential mechanisms contributing to GSH depletion in absence of hepatotoxicity. (i) The present APAP-induced increase in the expression ATF4-dependent gene Chac1, encoding the inducible glutathione-specific γ-glutamyl cyclotransferase, catalyzes the intracellular degradation of GSH. This is in favor of intracellular degradation of GSH in the liver of APAP-treated mice. Our result extends a single previous preliminary work reporting that overexpression of CHAC1 in HepG2 cells reduced GSH and increased APAP toxicity [28]. Further evaluation of the quantitative contribution of Chac1 to the APAP-induced GSH decrease is of interest because Chac1 could be a novel target to rescue hepatic GSH and reduce APAP toxicity. (ii) Our results also support that APAP-induced decrease in liver GSH could be due to impaired GSH synthesis. The liver responded to the low concentration of GSH by promoting the potential for GSH synthesis by increasing the mRNA expression of *Gclc* and *Gclm*. These ATF4-dependent genes encode the two subunits of γ-glutamyl-cysteine lyase, which catalyze the first step of GSH synthesis. Cys is recognized as the limiting amino acid for GSH synthesis [5]. This amino acid is synthesized from methionine and serine in the liver. Of note, *Psph* mRNA, encoding for phosphoserine phosphatase, which catalyzes the third and last step in the formation of serine [29] increased with APAP treatment. In the same line *Xct* mRNA, encoding for the inducible cystine transporter, was increased by the chronic treatment with APAP. All these transcriptional adaptations should provide more Cys and sustain the synthesis of GSH. Our present results show that liver GSH was low in APAP-treated mice. Hepatic GSH was recovered only when the plasma concentration of cyst(e)ine was restored by the dietary supplementation with Cys, meaning that the rate of GSH synthesis is limited by the APAP-induced Cys deficiency.

The amino acid sensor GCN2 detects the deprivation of a single essential amino acid and its activation upregulates the eIF2α-ATF4 signaling pathway. This activation elicits a transcriptional program of genes involved in the adaptation to amino acid deficiency (amino acid transport, amino acid synthesis…) [19]. We show here that (i) GCN2 is activated by APAP treatment inducing a low plasma concentration of cyst(e)ine and that (ii) this activation is abrogated by the dietary supplementation with Cys. Thus, the chronic treatment with APAP, at a dose equivalent to the maximum therapeutic dose for a human, increases the dietary requirement of cysteine and this amino acid becomes nutritionally essential. GCN2 activation is expected to play a role in the transcriptional response to chronic treatment with APAP, although its role is not critical due to overlap with the transcriptional response to the concomitant activation of PERK. Our data extend to the chronic treatment with a non-toxic dose of APAP, activation of the PERK-eIF2α-ATF4 signaling pathway observed after a single administration of a toxic dose of APAP [30]. PERK activation can presently result from either APAP-induced impairment of protein conformation in the endoplasmic reticulum due to the formation of APAP-protein adduct or oxidative alterations due to GSH depletion.

NRF2, the master transcription factor of the oxidative stress response interacts with ATF4 and cooperatively upregulates the expression of genes involved in mitochondrial quality control [31]. PERK has been shown to directly phosphorylate NRF2 in a mice cell line [32] and to activate Nrf2 transcription through ATF4 in a human cell line [33]. It has been shown that the lack of *Nrf2* increases the susceptibility of APAP toxicity through the increased formation of NAPQI due to limited capacity for APAP conjugation to sulfate or glucuronide [34]. Here, we show that chronic treatment with APAP increases NRF2 phosphorylation in the liver along with an increased transcription of *Nqo1*, a specific target of this transcription factor, and an increased transcription of *Xct*, *Gclc*, and *Gclm*, targets of both ATF4 and NRF2 [31,35]. Our results suggest that there is a cross-talk between ATF4 and NRF2 transcriptional responses to the chronic treatment with APAP in the liver.

Muscle wasting results from complex dysfunctions related to fiber renewal and/or protein turnover. Here we confirm that chronic treatment with APAP induces muscle wasting that is abrogated with the dietary supplementation with Cys. We report a moderate activation of the eIF2α-ATF4 signaling pathway in skeletal muscle from APAP-treated mice. ATF4 has been previously found to promote muscle wasting during fasting, immobilization, and aging in part by increasing the expression of *Gadd45a*, *p21*, and *Eif4ebp1* [15,16,36]. Here we show that Gadd45a mRNA was increased in the muscle of APAP-treated mice and that this increase was abolished with the dietary supplementation with Cys. Gadd45a was shown to be the centerpiece of the ATF4 signaling network that drives skeletal muscle atrophy [15,37]. However, the role of the eIF2α-ATF4 signaling pathway in muscle wasting induced by Cys deficiency consecutive to the chronic treatment with APAP remains to be demonstrated.

Our data also show that the eIF2α-ATF4 signaling pathway is activated in other organs such as the pancreas, kidney, small intestine, and brain. Further analysis using tissue-specific loss-of-function models will help to further explore the role of this pathway in the response to APAP treatment. In the hypothalamus, the eIF2α phosphorylation is involved in anorexia induced by a single amino acid deprivation [38]. However; the role of the eIF2α-ATF4 signaling pathway in the reduced food intake observed with the APAP diet remains to be explored. Overall, the eIF2α-ATF4 signaling pathway should have a broad role in the organism’s adaptation to chronic APAP treatment and therefore could be a privileged target in the fight against APAP-induced metabolic adverse effects.

## 4. Materials and Methods

### 4.1. Ethics Statement

Maintenance of the mice and all experiments were conducted according to the guidelines formulated by the European Community for the use of experimental animals (2010/63/EU). INRAE animal facilities were approved by the French veterinary department (IEN D6334515). Animal experiments were approved by the national regulatory committee of the Ministère de la l’Enseignement Supérieur de la Recherche et de l’Innovation (APAFIS#23636).

### 4.2. Animals

CARE-Luciferase (CARE-LUC) transgenic mice expressing the luciferase reporter gene under the control of the thymidine kinase promoter activated by two Trb3 (tribbles pseudokinase 3) sequences containing the three CARE (C/EBP (CCAAT/enhancer-binding protein)-ATF response element) sequences of recognition were previously described [18]. The generation of *Gcn2* null mice has been described in detail elsewhere [39,40]. Mice were maintained in our animal facility in a temperature-controlled room (22 ± 1 °C) on a 12/12h light-dark cycle. They were provided free access to commercial rodent chow (pellets A03 from Safe, Augy, France) and tap water prior to the experiment.

### 4.3. APAP Treatment and Experimental Diets

APAP was administered within the diet. Six experimental powdered diets (Table S1) were manufactured in our institute facilities (INRAE, Unité de Préparation des Aliments Expérimentaux, Jouy-en-Josas, France). The control diet (Ctrl diet) was a semi-synthetic diet with 16% free L-amino acids. APAP powder (Sigma-Aldrich, St. Louis, MO, USA) was mixed with the ingredients of the Crtl diet (1% (*w/w*) APAP diet), as an equivalent to the maximum therapeutic dose for humans [4]. To study the effect of the dietary supplementation with Cys on the response to APAP treatment, the Ctrl diet and APAP diet were supplemented with 0.5% L-cysteine (Cys diet and APAP–Cys diet, respectively). The Cys supplement aimed to cover the extra need of Cys needed to detoxify APAP [20]. The Ctrl and APAP diets were supplemented with L-alanine (0.37%) to make them iso-nitrogenous to Cys diet and APAP–Cys diet (Ala diet and APAP–Ala diet, respectively). Experimental diets were administered *ad libitum* or in pair-feeding for 18 d, as mentioned above. The 6 h time point was preceded by a 16 h fasting period to synchronize the consumption of all mice. Experiments were performed by using a minimum of six animals per group. Mice were placed in individual cages allowing for daily food intake measurement.

### 4.4. Bioluminescence Imaging

Bioluminescence imaging was performed using the IVIS Spectrum (PerkinElmer, Villebon-sur-Yvette, France) imaging scanner coupled to the Living Image Software (Perkin Elmer) on IVIA multimodal imaging platform (Clermont-Ferrand, France) (https://doi.org/10.18145/ivia accessed on 17 May 2022). Briefly, 100 mg/kg of in vivo luciferase substrate (beetle luciferin substrate, Promega) were injected intraperitoneally into each CARE-LUC mouse. Mice were then anesthetized with isoflurane and scanned 10 min after luciferin injection. The abdominal cavity was shaved to allow for the accurate collection of bioluminescence signals. Light emission was quantified with imaging software from constant regions of interest (ROI) drawn manually. Photon emission was measured as radiance in p·s^−1^·cm^−2^·sr^−1^. The sensitivity of the imaging scanner was tested weekly with commercially available positive sources of bioluminescence. Excised organs were imaged immediately after euthanasia using the IVIS Spectrum (PerkinElmer) imaging scanner coupled to the Living Image software (PerkinElmer).

### 4.5. Luciferase and GSH Biochemical Assays

Luciferase assays were performed in tissue extracts as previously described [14]. Luciferase activities measured were normalized to protein content (relative light units per milligram of protein) and expressed as fold induction relative to the control condition. Total free GSH (GSH, GSSG, and other small disulphides) concentration was quantified in the liver and TA muscle with an automated analyzer (ABX Pentra 400; Horiba, Kyoto, Japan) using a standard enzymatic recycling procedure and 5,5′-dithio-bis-2-nitrobenzoic acid (Ellman reagent) as oxidant, as previously described [41].

### 4.6. Plasma Biochemical Analyses

Blood was withdrawn by intracardiac puncture onto EDTA, plasma was immediately collected and frozen, then kept at −80 °C until analysis.

Hepatotoxicity was assessed by measurement of plasma alanine transaminase (ALT) and aspartate transaminase (AST) activities by photometry using an automated analyzer (ABX Pentra 400) and test kits A11A01629 and A11A01627 (Horiba), respectively.

Free cyst(e)ine (reduced form + homodimer + small heterodimers) was quantified by reversed-phase HPLC conditions and fluorescence detection [42]. Briefly, plasma proteins were precipitated with trichloroacetic acid and supernatant treated with tris-(2-carboxyethyl)-phosphine hydrochloride to reduce cyst(e)ine. Cys was derivatized with amonim-7-fluorobenzo-2-oxa-1,3-diazole-4-sulfonic acid. An Alliance HPLC system (Waters, Guyancourt, France) was equipped with a hypersil gold column (4.6 × 150 mm, 3 μm, Thermo Scientific, Illkirch, France). The two mobile phases consisted of A: 0.1 M acetate buffer (pH 4.5)—methanol 97:3 (*v/v*) and B: buffer A—methanol 78:22 (*v/v*). Aminothiols were separated over 2 min with 100% A at a flow rate of 1 mL·min^–1^, followed by 100% B (changed linearly over 1 min) at a flow rate of 1.0 mL·min^–1^ for 7 min, and 5 min of column re-equilibration for a total run time of 16 min.

### 4.7. Analysis of Gene Expression Using Real-Time RT-qPCR

Total RNA was prepared using a phenol-chloroform extraction followed by a purification with NucleoSpin RNA mini kit (Macherey Nagel, Düren, Germany) and treatment with DNase I, Amp Grade (Invitrogen, Carlsbad, CA, USA). RNA (0.5 µg) was reverse transcribed with 100 U of Superscript II plus RNase HReverse Transcriptase (Invitrogen) using 100 mM random hexamer primers (Amersham Biosciences, Piscataway, NJ, USA), according to the manufacturer’s instructions. Real-time quantitative PCR was carried out on a Bio-Rad CFX-96 detection system with quantitative qPCR SYBR Green reagents (Bio-Rad, Hercules, CA, USA) and with a primer concentration of 0.5 mM. PCR conditions were standardized to 39 cycles of 95 °C for 10 s, 59 °C for 10 s, and 72 °C for 20 s with the primers for specific mouse mRNA sequences. Primers for mouse sequences (Table S2) yielded PCR products of approximatively 100–150 bp in size. To control for RNA quality and cDNA synthesis, *Gapdh* mRNA was used. The abundance of each mRNA was normalized to the *Gapdh* signal. Each experiment was repeated three times to confirm the reproducibility of the results.

RNA integrity was electrophoretically verified by ethidium bromide staining. RNA (0.5 µg) was reverse transcribed with 100 U of Superscript II plus RNase HReverse Transcriptase (Invitrogen) using 100 mM random hexamer primers (Amersham Biosciences, Piscataway, NJ, USA), according to the manufacturer’s instructions. Real-time quantitative PCR was carried out on a Bio-Rad CFX-96 detection system with quantitative qPCR SYBR Green reagents (Bio-Rad, Hercules, CA, USA) and with a primer concentration of 0.5 mM. PCR conditions were standardized to 40 cycles of 95 °C for 10 s and 59 °C for 30 s with the primers for specific mouse mRNA sequences. Primers for mouse sequences (Table S2) yielded PCR products of approximatively 100–150 bp in size. To control for RNA quality and cDNA synthesis, *Gapdh* mRNA was used. The abundance of each mRNA was normalized to the *Gapdh* signal. Each experiment was repeated three times to confirm the reproducibility of the results.

### 4.8. Immunoblot Analysis

Whole proteins were extracted from 10 mg of liver with 500 µL of extraction buffer (20 mM HEPES pH 7.4, 100 mM KCl, 0.2 mM EDTA, 2 mM EGTA, 1 mM DTT, 1%Triton, 50 mM β-glycerophosphate, protease and phosphatase inhibitor cocktail from Sigma). Proteins were separated by SDS-polyacrylamide gel electrophoresis and transferred onto a Hybond-P PVDF membrane (Amersham Biosciences). Membranes were blocked for 1 h at room temperature with a solution of 5% nonfat milk powder in TBST (50 mM Tris-HCL, pH 8.0, 150 mM NaCl, 0.1% Tween-20). The blots were then incubated with an antibody in a blocking solution overnight at 4 °C. Antibodies were diluted according to the manufacturer’s instructions. The blots were washed three times in TN and incubated with horseradish peroxidase-conjugated goat anti-IgG (1/2000) (Cell Signaling) in a blocking buffer for 1 h at room temperature. After three washes, the blots were developed using the LuminataTM Western HRP substrate (Millipore, Billerica, MA, USA).

### 4.9. Antibodies

ATF4 (# 11815), GCN2 (# 3302), eIF2α (# 9722), NRF2 (# 12721), PERK (#3192) and normal rabbit IgG (# 7074) antibodies were purchased from Cell Signaling Technology (Beverly, MA). P-GCN2 (ab75836), P-NRF2(ab76026) and P-eIF2α (ab32157) antibodies was obtained from Abcam (Cambridge, UK). GAPDH (G9545) antibody was from Sigma-Aldrich (Saint Louis, MO, USA).

### 4.10. Cross-Sectional Area of Muscle Fibers

Muscle cross-sections (10 µm thick) were obtained using a cryostat (HM500M Microm International, Fisher Scientific, Illkirch, France) at −20 °C. Cross-sections were labeled with anti-laminin-α1 (L9393 Sigma, Saint-Quentin-Fallavier, France) to outline the fibers, and resolved with corresponding secondary antibodies conjugated to Alexa-Fluor 488 (Invitrogen, Cergy-Pontoise, France). Observations and image acquisitions were captured with a high-resolution ORCA-Flash4.0 LT+ Digital CMOS camera coupled to an IX-73 microscope (Olympus) and Cell-Sens dimension software (Olympus Soft Imaging Solutions, Münster, Germany). The cross-sectional area (CSA) was determined for each fiber, using ImageJ 1.53f51 (http://rsb.info.nih.gov/ij/ accessed on 17 May 2022).

### 4.11. Statistical Analysis

A minimum of six mice were used for each group. Data are expressed as means ± SEM or as median with an interquartile range as mentioned. The significance of differences was analyzed by Student’s *t*-test or Mann-Whitney test, respectively. Analyses were performed using GraphPad Prism 9.3.1 and the significance was set at *p* ≤ 0.05.

## Data Availability

Not applicable.

## Supplementary Materials

The following supporting information can be downloaded at: https://www.mdpi.com/article/10.3390/ijms23137196/s1.
