# Supplementary material for: Activation of the eIF2α-ATF4 Pathway by Chronic Paracetamol Treatment Is Prevented by Dietary Supplementation with Cysteine
Source: Int J Mol Sci. 2022 Jun 28;23(13):7196. doi: 10.3390/ijms23137196 (PMC9266523; doi:10.3390/ijms23137196)
Supplement: Supplementary file 1 [file ijms-23-07196-s001.zip › ijms-1763192-supplementary.pdf]

Table S1: Composition of the experimental diets

	Ctrl	APAP	Ala	APAP−Ala	Cys	APAP−Cys
L-alanine	5.5	5.5	9.2	9.2	5.5	5.5
L-arginine	8.5	8.5	8.5	8.5	8.5	8.5
L-asparagine	6.5	6.5	6.5	6.5	6.5	6.5
L-aspartate	6.0	6.0	6.0	6.0	6.0	6.0
L-cysteine	-	-	-	-	5.0	5.0
L-cystine	2.5	2.5	2.5	2.5	2.5	2.5
L-glutamine	11.5	11.5	11.5	11.5	11.5	11.5
L-glutamate	20.5	20.5	20.5	20.5	20.5	20.5
L-glycine	5.0	5.0	5.0	5.0	5.0	5.0
L-histidine	5.0	5.0	5.0	5.0	5.0	5.0
L-isoleucine	7.5	7.5	7.5	7.5	7.5	7.5
L-leucine	14.5	14.5	14.5	14.5	14.5	14.5
L-lysine	16.5	16.5	16.5	16.5	16.5	16.5
L-methionine	2.5	2.5	2.5	2.5	2.5	2.5
L-phenylalanine	8.0	8.0	8.0	8.0	8.0	8.0
L-proline	11.0	11.0	11.0	11.0	11.0	11.0
L-serine	6.0	6.0	6.0	6.0	6.0	6.0
L-threonine	7.0	7.0	7.0	7.0	7.0	7.0
L-tryptophan	2.0	2.0	2.0	2.0	2.0	2.0
L-tyrosine	4.5	4.5	4.5	4.5	4.5	4.5
L-valine	9.5	9.5	9.5	9.5	9.5	9.5
Starch	552.5	542.5	548.8	538.8	547.5	537.5
Sucrose	150	150	150	150	150	150
Agar-agar	30.0	30.0	30.0	30.0	30.0	30.0
Rapeseed oil	30.0	30.0	30.0	30.0	30.0	30.0
Peanut oil	24.0	24.0	24.0	24.0	24.0	24.0
Sunflower oil	6.0	6.0	6.0	6.0	6.0	6.0
AIN-93 Mineral mix	35.0	35.0	35.0	35.0	35.0	35.0
AIN-93 Vitamin mix	10.0	10.0	10.0	10.0	10.0	10.0
Choline bitartrate	2.5	2.5	2.5	2.5	2.5	2.5
APAP	-	10.0	-	10.0	-	10.0

Ala: Control diet supplemented with L-alanine; APAP: acetaminophen; APAP−Ala: APAP diet supplemented with L-alanine; APAP−Cys: APAP diet supplemented with L-cysteine Ctrl: control diet; Cys: Ctrl diet supplemented with L-cysteine.

Table S2: List of primers sequences used for RT-qPCR analysis in this study

**Gene**	**Forward primer**	**Reverse primer**
Asns	5′-TACAACCACAAGGCGCTACA-3′	5′-AAGGGCCTGACTCCATAGGT-3′
Atf6	5′-TTCTGGGAGTGAGCTGCAAG-3′	5′-TTGTTGTGGGTGGTAGCTGG-3′
Chac1	5′-CATAGGGGCAGCGACAAGATG-3′	5′-CTGTGTGGCAATGACCTCTTC-3′
Chop	5′-CCTAGCTTGGCTGACAGAGG-3′	5′-CTGCTCCTTCTCCTTCATGC-3′
Gadd45a	5′-AGTCAACTTATTTGTTTTTGC-3′	5′-GCAATTTGGTTCAGTTATTT-3′
Gapdh	5′-TCGGAGTCAACGGATTTGGT-3′	5′-TGGAATTTGCCATGGGTGGAA-3′
Gclc	5′-ATGTGGACACCCGATGCAGTATT-3′	5′-TGTCTTGCTTGTAGTCAGGATGGTTT-3′
Gclm	5′-GCCACCAGATTTGACTGCCTTT-3′	5′-CAGGGATGCTTTCTTGAAGAGCTT-3′
Grp78	5′-ATTGGAGGTGGGCAAACCAA-3′	5′-TCGCTGGGCATCATTGAAGT-3′
Nqo1	5′-CATTGCAGTGGTTTGGGGTG-3′	5′-TCTGGAAAGGACCGTTGTCG-3′
Psph	5′-CCAATAGGCTGAAGTTCTAC-3′	5′-TAAAAACCGAATAACCTTTC-3 ′
Trb3	5′-CCAGAGATACTCAGCTCCCG-3′	5′-GAGGAGACAGCGGATCAGAC-3′
Xbp1t	5′-TACGGGAGAAAACTCACGGC-3′	5′-AAACCTGCAGCCAGGTAGTG-3′
Xbp1s	5′-CTGACGAGGTTCCAGAGGTG-3′	5′-ACATAGTCTGAGTGCTGCGG-3′
Xct	5′-GCATGTCCCTGGTTTTCTGG-3′	5′-AAGCCAGCAAAGGACCAAAG-3′

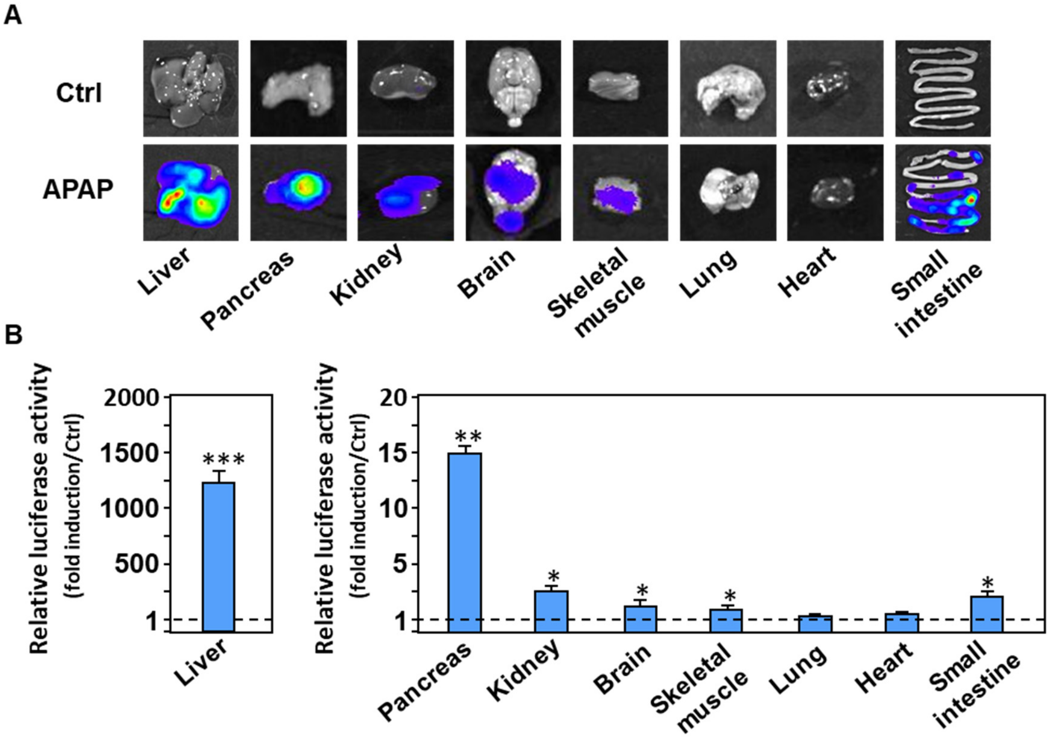

Figure S1. Identification of the eIF2α-ATF4 pathway target tissues in response to 8 d of APAP treatment. (**A**) Visualization of bioluminescence by imaging of several organs. CARE-LUC mice were fed the control diet (Ctrl) or the APAP diet (APAP) for 8 d. After bioluminescence imaging, mice were sacrificed by cervical dislocation, organs were rapidly excised and imaged, followed by snap-freezing of the organ for subsequent luciferase enzyme assay. (**B**) Luciferase activity measured in tissue extracts. Results are given as fold induction in APAP group relative to Ctrl group. The graphs show means ± S.E.M. of 6 mice. * $P \leq 0.05$; ** $P \leq 0.01$; *** $P \leq 0.001$ compared to the respective Ctrl value, Student's t test.

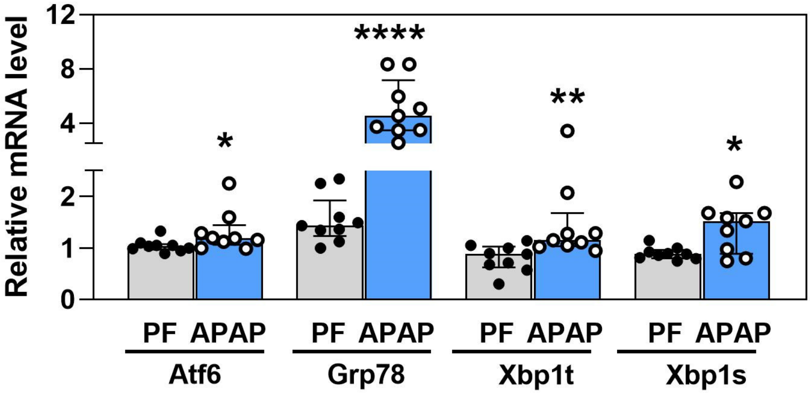

Figure S2. Effect of APAP treatment on unfolded protein response (UPR) marker genes in the liver. Mice were fed a diet with 1% APAP (APAP) for 18 d or were pair-fed (PF) the control diet to match the daily food intake of the respective APAP-treated mice. Total RNA extracted from liver was analyzed by RT-qPCR. Each bar shows the median with interquartile and dots are individual values. * *P* ≤0.05; ** *P* ≤0.01; *** *P* ≤0.001 *vs* PF; Mann Whitney test.

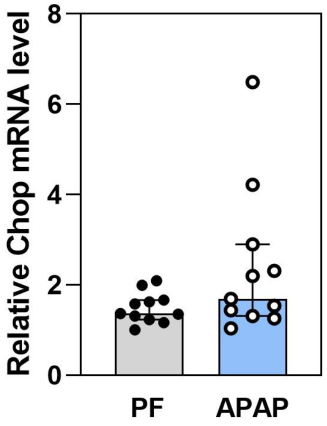

Figure S3. Expression of *Chop* in the liver of mice treated with APAP for 18 d. Mice were fed a diet with 1% APAP (APAP) for 18 d or were pair-fed (PF) the control diet to match the daily food intake of the respective APAP-treated mice. Total RNA extracted from liver was analyzed by RT-qPCR. Each bar shows the median with interquartile and dots are individual values. No significant effect (Mann-Whitney test).
